# Supplementary material for: Harmonization of Biobank Education for Biobank Technicians: Identification of Learning Objectives
Source: BioTech (Basel). 2021 Apr 14;10(2):7. doi: 10.3390/biotech10020007 (PMC9336536; doi:10.3390/biotech10020007)
Supplement: Supplementary file 1 [file biotech-10-00007-s001.zip › Table S1.pdf]

Supplemental Table S1: Questionnaire for the co-workers of the technical personnel

Relevant demographics		
No.	*Question*	*Answer choices*
1	Your position with the biobank?	Technical personnel<br>Biobank director<br>Project/ biobank management<br>Laboratory head<br>Quality management<br>IT co-worker
2	How many years have you been working in the biobank?	*Free text field*
3	How many technical staff work in your biobank?	*Free text field*
4	How good do you think are the English skills of most of your technical staff?	Native speaker<br>They can communicate easily in English about technical content and understand texts on the first try.<br>They can communicate in English.<br>Their English is rather weak.<br>They can't communicate in English at all.
**Questions concerning the educational needs of the technical personnel**		
No.	*Question*	*Answer choices*
5.a	How many of your technical staff would benefit from further training in "collection and transport of biospecimens"?	All<br>Most<br>Half<br>Few<br>None
5.b	Which topic in this area is particularly important for you?	*Free text field*
6.a	How many of your technical staff would benefit from further training in "entry, processing, and release of biospecimens"?	All<br>Most<br>Half<br>Few<br>None
6.b	Which topic in this area is particularly important for you?	*Free text field*
7.a	How many of your technical staff would benefit from further training in "storage of biospecimens"?	All<br>Most<br>Half<br>Few<br>None
7.b	Which topic in this area is particularly important for you?	*Free text field*
8.a	How many of your technical staff would benefit from further training in "quality management"?	All<br>Most<br>Half<br>Few<br>None
8.b	Which topic in this area is particularly important for you?	*Free text field*
9.a	How many of your technical staff would benefit from further training in "data management and data protection"?	All<br>Most<br>Half

		Few
		None
9.b	Which topic in this area is particularly important for you?	*Free text field*
10.a	How many of your technical staff would benefit from further training in the area of "occupational safety"?	All
		Most
		Half
		Few
		None
10.b	Which topic in this area is particularly important for you?	*Free text field*
11.a	How many of your technical staff would benefit from further training in the area of "ethical, legal and social issues"?	All
		Most
		Half
		Few
		None
11.b	Which topic in this area is particularly important for you?	*Free text field*
12	If you have other topics that you consider important with regards to further trainings, you can list them here:	*Free text field*
13	For which of the following areas do you think your technical staff need practical skills? (Multiple answers possible)	Collection and transport of biospecimens
		Entry, processing, and release of biospecimens
		Storage of biospecimens
		Quality management
		Data management and data protection
		Occupational safety
		Ethical, legal, and social issues
		Others (*free text field*)
14	Which of the following areas should be supported by practical training measures? (Multiple answers possible)	Collection and transport of biospecimens
		Entry, processing, and release of biospecimens
		Storage of biospecimens
		Quality management
		Data management and data protection
		Occupational safety
		Ethical, legal, and social issues
		Other (*free text field*)
15	In which areas do you think your technical staff only needs basic knowledge? (Multiple answers possible)	Collection and transport of biospecimens
		Entry, processing, and release of biospecimens
		Storage of biospecimens
		Quality management
		Data management and data protection
		Occupational safety
		Ethical, legal, and social issues
		Other (*free text field*)

Assessment of already existing knowledge		
No.	***Question***	***Answer choices***
16	My technical staff understood what the main task of a biobank is.	Yes
		No
	Please assess the skills and knowledge of your technical staff on the learning objectives below:	
17	They can explain the importance of preanalytical factors for the quality of biospecimens and can describe how to document them.	
18	They can describe the different types of biospecimens.	

19	They know for which analyses FFPE and cryogenic tissue are suitable or not suitable.	*7-item likert scale:*<br>○ Strongly agree<br>○<br>○<br>○<br>○<br>○<br>○ Strongly disagree
20	They can describe the necessary steps needed for the transport of biospecimens.	
21	They know how to document the entry and release of biospecimens.	
22	They can explain different processing methods for biospecimens.	
23	They can describe the most important aspects of processing and storage of biospecimens.	
24	They know how to prepare various biospecimens for shipment (e.g., by courier transport).	
25	They can describe how biospecimens should be handled best for further processing after storage.	
26	They can describe how to store and retrieve biospecimens to ensure their optimal use.	
27	They know how to handle stored biospecimens.	
28	They know how to proper process cryospecimens without thawing them.	
29	They can document deviations from SOPs.	
30	You know the difference between anonymization and pseudonymization.	
31	They can explain the purpose of the “Material and Data Transfer Agreement”.	
32	They know how to dispose biospecimens that are not going to be used.	
33	They know how to proceed with stored biospecimens in the event of a revocation of consent.	
34	They know how to handle liquid nitrogen and dry ice.	
35	They how to react in a case of an accident.	

Experience with further trainings

No.	*Question*	*Answer choices*
36	Did your technical staff already participate in further training?	Yes, all
		Yes, some
		None
37	On how many training courses did your technical staff participate?	○ None<br>○ 1<br>○ 2<br>○ 3<br>○ 4<br>○ 5<br>○ 6<br>○ 7<br>○ 8<br>○ 9<br>○ 10<br>○ More than 10
38	What was the topic of the training?	*Free text field*
39	Was the training specifically for technical staff?	Yes
		No
40	What was the format of the training?	Practical course
		Online- course
		Lecture/ theory/ seminar
		eLearning
		Others (*free text field*)
41	How long did the training last?	1 to less than 3 hours

		3 to less than 5 hours
		5 to 8 hours
		1 - 2 days
		3 – 5 days
		Longer
42	In which environment did the training take place?	Academy
		Industry
		Others (*free text field*)
43	How much did the training cost per employee? (in €)	*Free text field*
44	How motivated was your technical staff to participate in the training?	*7-item Likert scale:*<br>o Absolutely not motivated<br>o<br>o<br>o<br>o<br>o<br>o Highly motivated
45	How satisfied were your technical staff with the training?	*7-item Likert scale:*<br>o Absolutely not satisfied<br>o<br>o<br>o<br>o<br>o<br>o Very satisfied

Questions concerning the educational needs of the technical personnel

No.	***Question***	***Answer choices***
46	Do you have any suggestions and proposals on how to improve further training measures?	*Free text field*
47	Have you already had experience with eLearning courses?	Yes
		No
48	What do you think are the advantages of eLearning?	*Free text field*
49	What do you think are the disadvantages of eLearning?	*Free text field*
50	Which learning method do you prefer?	Printed scripts
		eLearning/ online course
		Lecture/ theory/ seminar
		Practical course
		Combination of theoretical and practical training
51	Would you appreciate a final test in a further training measure to examine the learning success of your technical staff?	Yes
		No
52	Would you appreciate a certificate being issued to your technical staff as proof that they passed the test?	Yes
		No
53	What budget would be available for further training? (in € per year)	*Free text field*
54	In my opinion, a good further training measure should:	… be well organized
		… communicate knowledge in a simple and understandable way
		… take place in groups
		… be designed for self-study

		… be application-oriented
		Other (*free text field*)
**Relevant demographics**		
No.	***Question***	***Answer choices***
55	What age group are you in?	younger than 20
		20-29
		30-39
		40-49
		50-59
		60 and older
56	Is there anything else you would like to tell us concerning our survey or the topic of "Further training in biobanks"? Here you have the opportunity to do so:	*Free text field*
