# Supplementary material for: Harmonization of Biobank Education for Biobank Technicians: Identification of Learning Objectives
Source: BioTech (Basel). 2021 Apr 14;10(2):7. doi: 10.3390/biotech10020007 (PMC9336536; doi:10.3390/biotech10020007)
Supplement: Supplementary file 1 [file biotech-10-00007-s001.zip › Table S2.pdf]

Supplementary Table S2: Questionnaire for the technical personnel.

**Relevant demographics**		
No.	***Question***	***Answer choices***
1	Your position with the biobank?	Technical personnel<br>Biobank director<br>Project/ biobank management<br>Laboratory head<br>Quality management<br>IT co-worker
2	What kind of education do you have as technical personnel?	Medical technical assistant<br>Biological technical assistant<br>Laboratory technician<br>Another educational qualification, specifically: (*free text field*)
3	For how many years have you been working with the biobank?	Less than 1 year<br>1 to less than 3 years<br>3 to less than 5 years<br>5 years and longer
4	What describes your English skills best?	I am a native speaker<br>I can communicate easily in English on technical subjects and understand texts straight away<br>I can communicate in English<br>My English is rather weak<br>I cannot speak English at all
**Educational needs**		
No.	***Question***	***Answer choices***
	In which areas would you like to have further training?	
5.a	Collection and transport of biospecimens	*7-item likert scale:*<br>o Absolutely not interested<br>o<br>o<br>o<br>o<br>o<br>o Very interested
5.b	Which topic in this area is particularly important for you?	*Free text field*
6.a	Entry, processing, and release of biospecimens	*7-item likert scale:*<br>o Absolutely not interested<br>o<br>o<br>o<br>o<br>o<br>o Very interested
6.b	Which topic in this area is particularly important for you?	*Free text field*
7.a	Storage of biospecimens	*7-item likert scale:*<br>o Absolutely not interested<br>o<br>o<br>o<br>o<br>o<br>o Very interested

7.b	Which topic in this area is particularly important for you?	*Free text field*
8.a	Quality management	*7-item likert scale:*<br>○ Absolutely not interested<br>○<br>○<br>○<br>○<br>○<br>○ Very interested
8.b	Which topic in this area is particularly important for you?	*Free text field*
9.a	Data management and data protection	*7-item likert scale:*<br>○ Absolutely not interested<br>○<br>○<br>○<br>○<br>○<br>○ Very interested
9.b	Which topic in this area is particularly important for you?	*Free text field*
10.a	Occupational Safety	*7-item likert scale:*<br>○ Absolutely not interested<br>○<br>○<br>○<br>○<br>○<br>○ Very interested
10.b	Which topic in this area is particularly important for you?	*Free text field*
11.a	Ethical, legal, and social issues	*7-item likert scale:*<br>○ Absolutely not interested<br>○<br>○<br>○<br>○<br>○<br>○ Very interested
11.b	Which topic in this area is particularly important for you?	*Free text field*
12	If there are other topics from your point of view that are important to you in connection with further education, you can enter them here:	*Free text field*
13	For which of these areas do you need practical skills? (Multiple answers possible)	Collection and transport of biospecimens<br>Entry, processing, and release of biospecimens<br>Storage of biospecimens<br>Quality management<br>Data management and data protection<br>Occupational safety<br>Ethical, legal, and social issues<br>Other (*free text field*)
14	Which of these areas should be supported by practical exercises in a further training measure? (Multiple answers possible)	Collection and transport of biospecimens<br>Entry, processing, and release of biospecimens<br>Storage of biospecimens<br>Quality management

		Data management and data protection
		Occupational safety
		Ethical, legal, and social issues
		Other (*free text field*)
15	In which of these areas is basic knowledge sufficient? (Multiple answers possible)	Collection and transport of biospecimens
		Entry, processing, and release of biospecimens
		Storage of biospecimens
		Quality management
		Data management and data protection
		Occupational safety
		Ethical, legal, and social issues
		Other (*free text field*)

**Existing knowledge**		
No.	***Question***	***Answer choices***
16	I know what the main task of a biobank is.	Yes
		No
	Please assess your skills and knowledge on the following learning objectives below:	
17	I can explain the importance of pre-analytical factors for the quality of biospecimens and can describe how these shall be documented.	*7-item likert scale:*<br>○ Disagree entirely<br>○<br>○<br>○<br>○<br>○<br>○ Agree entirely
18	I can describe different types of biospecimens.	
19	I know for which analyses FFPE and cryogenic tissue are suitable or not suitable.	
20	I can describe the necessary steps for the transport of biospecimens.	
21	I know how to document entry and release of biospecimens.	
22	I can explain different processing methods for biospecimens.	
23	I can describe important aspects of biospecimens processing and storage.	
24	I know how to prepare different biospecimens for shipment (e.g. by courier transport).	
25	I can describe how the treat biospecimens after storage for further processing.	
26	I can describe how to store and retrieve biospecimens to ensure their optimal use.	
27	I know how to handle stored biospecimens.	
28	I know how to properly process cryospecimens without thawing them.	
29	I can document deviations from the SOP.	
30	I know the difference between anonymization and pseudonymization	
31	I can explain the purpose of the "Material and Data Transfer Agreement".	
32	I know how to dispose biospecimens that are not going to be used.	
33	I know what to do with stored biospecimens in the event of a revocation of consent.	
34	I know how to handle liquid nitrogen and dry ice.	
35	I know how to react in a case of an accident.	

**Previous participation in advanced trainings**		
No.	***Question***	***Answer choices***
36	In how many different training courses did you participate ?	None
		1
		2
		3
		4
37	What was the topic of the training?	*Free text field*
38	Was the training specifically for technical staff?	Yes

		No
39	What was the format of the training?	Practical course<br>Online course<br>Lecture/ theory/ seminar<br>eLearning<br>Other (*free text field*)
40	How long did the training last?	1 to under 3 hours<br>3 to under 5 hours<br>5 to 8 hours<br>1 to 2 days<br>3 to 5 days<br>Longer
41	In which environment did the training take place?	Academy<br>Industry<br>Other (*free text field*)
42	How much did it cost? (in €)	*Free text field*
43	How difficult was it for you to get time off for the training?	*7 -item likert scale:*<br>o Very difficult<br>o<br>o<br>o<br>o<br>o<br>o Not difficult at all
44	How motivated were you to participate in the training?	*7-item Likert scale:*<br>o Absolutely not motivated<br>o<br>o<br>o<br>o<br>o<br>o Highly motivated
45	How satisfied were you with the training?	*7-item Likert scale:*<br>o Absolutely not satisfied<br>o<br>o<br>o<br>o<br>o<br>o Very satisfied
**Questions along the design of a future advanced training for biobank technicians**		
No.	***Question***	***Answer choices***
46	Do you have any suggestions and proposals on how to improve further training measures?	*Free text field*
47	Have you already had experience with eLearning courses?	Yes<br>No
48	What do you think are the advantages of eLearning?	*Free text field*
49	What do you think are the disadvantages of eLearning?	*Free text field*
50	What kind of learning method do you prefer?	eLearning/ online course<br>Printed scripts<br>Lecture/ theory/ seminar<br>Practical course<br>Combination of theoretical and practical training<br>Other, please fill in: (free text field)

51	Would you appreciate a final test in a further training measure to check your learning success?	Yes<br>No
52	Would you like to receive a certificate as proof that you passed the examination?	Yes<br>No
53	In my opinion, a good further education measure should:	… be well organized<br>… communicate knowledge in a simple and understandable way<br>… take place in groups<br>… be designed for self-study<br>… be application-oriented<br>Other (free text field)
**Ending of the survey**		
No.	***Question***	***Answer choices***
54	What age group are you in?	younger than 20<br>20-29<br>30-39<br>40-49<br>50-59<br>60 and older
55	Is there anything else you would like to tell us concerning our survey or the topic of "Further training in biobanks"? Here you have the opportunity to do so:	*Free text field*
