# Supplementary material for: Harmonization of Biobank Education for Biobank Technicians: Identification of Learning Objectives
Source: BioTech (Basel). 2021 Apr 14;10(2):7. doi: 10.3390/biotech10020007 (PMC9336536; doi:10.3390/biotech10020007)
Supplement: Supplementary file 1 [file biotech-10-00007-s001.zip › Table S3.pdf]

Supplementary Table S3: Specific topics of interest for advanced training stated by technical personnel and co-workers

**Collection and transport of biospecimens**	
Technical personnel	Co-Workers of the technical personnel
• Transport conditions and documentation<br>• Optimal conditions for the different biospecimens (confounding factors e.g., CO_2 influence, N_2 without crystal formation)<br>• Release<br>• Transporting nitrogen containers<br>• Release<br>• Improve stability of RNA and DNA<br>• On-time collection of samples from the collection site<br>• Information on sample collection, further processing of the biospecimens in the clinic	• Practical proposals for solutions, i.e. technical implementation<br>• Sensitization with regards to the importance of all processes in biobanking, including release and transportation<br>• Time recording of release / transport<br>• Link between biobank and Clinic/ Study Centre<br>• Focus on biospecimens quality and know and follow formal rules<br>• Parameters that should be considered from the time of collection, sources of error<br>• Sensitive parameters, sources of error, aliquot size, temperature<br>• General, overarching standards for biobanks<br>• Avoid thawing of the sample<br>• Comparison of own processes to processes and standards in other biobanks<br>• Quality aspects, documentation aspects and occupational safety<br>• Reliability, quality awareness, discretion<br>• Release: Assisting in outpatient clinics and in the operating theatre<br>• Consistent cooling chain
**Entry, processing, and release of biospecimens**	
Technical personnel	Co-Workers of the technical personnel
• Material Transfer Agreement<br>• Documentation<br>• Simplification of workflows<br>• How samples are correctly released<br>• Processes and regulations<br>• To work precisely<br>• Avoidance of error sources<br>• Databases<br>• A good software	• Transfer of ownership<br>• Regulations<br>• Focus on biospecimens quality and knowledge and observance of formal regulations<br>• Parameters that must be considered; sources of error<br>• Sensitive parameters, sources of error, temperature<br>• Good documentation<br>• Quality aspects, documentation aspects, and occupational safety. In addition ,an exchange of best practices between technical staff at the various locations. Concerning release of biospecimens, if the quality is measured, it would be interesting to talk about thawing protocols.<br>• Work according to QM<br>• Critical analysis of the own work
**Storage of biospecimens**	

Technical personnel	Co-Workers of the technical personnel
• Cryotank expertise<br>• Quality assurance<br>• Long-term storage, quality control<br>• Influence of temperature on biospecimens<br>• Improve stability of RNA and DNA<br>• That the temperature is correct<br>• Labelling & storage systems<br>• Storage of biospecimens	• Overview of storage options in combination with short background information (e.g., automation vs. non-automation, different storage options) deep-frozen temperature. - which biospecimens)<br>• Handling of machines (troubleshooting)<br>• Redistribution of samples<br>• Focus on the quality of biospecimens and the biochemical and biophysical background e.g., of temperature fluctuations and their influence on molecule movement, etc.<br>• Parameters that need to be considered; sources of error; temperature issues; what storage options are available<br>• Temperature, storage options (vessel, automation yes/no, trouble shooting)<br>• General, overarching standards for biobanks<br>• Quality control, freeze-thaw<br>• What is most important for which storage condition (nitrogen, automated at -80°C)<br>• Redistribution, maintenance -80°C<br>• Which vessels are suitable for which type of storage condition?
**Quality management (QM)**	
Technical personnel	Co-Workers of the technical personnel
• Development of SOPs<br>• Storage<br>• Processing and storage<br>• Workflow<br>• Procedures	• Importance of quality management, practical trainings.<br>• Overview of the topic quality management / processes<br>• Efficiency, Continual Improvement Process<br>• Structured QM<br>• Basic knowledge in QM, specifically in the field of biobanks<br>• Aliquot volume, storage temperature, number of freeze-thaw cycles, residual treatment<br>• General, overarching standards for biobanks<br>• Application of QM procedures, development of SOPs<br>• How to organize and document QM effectively<br>• QM topics relevant for the technical personnel. Concerning the direct handling of samples<br>• Transparency of processes
**Occupational safety**	
Technical personnel	Co-Workers of the technical personnel

<table>
<tr><td>Handling / working with nitrogen
Following the guidelines
First aid courses</td><td>Takes place within the annual laboratory instructions
Pathogens
Have basic knowledge and be aware of this in the daily working routine
Work on dry ice, gas phase liquid nitrogen
General, overarching standards for biobanks
Handling potentially infectious material
Work with dry ice, liquid nitrogen, above tanks
Mainly concerns nitrogen and its hazardousness and the risk of infection posed by samples.
Protection for yourself and others</td></tr>
<tr><td colspan="2">Ethical, legal, and social issues</td></tr>
<tr><td>Technical personnel</td><td>Co-Workers of the technical personnel</td></tr>
<tr><td>Multiple participation in studies
Basics
How is an ethics vote developed/approved?
Data protection
Approval of ethics votes</td><td>Rough overview of the framework conditions, sensitization to the "value" of samples and patent rights.
Ethics
Interface between biobank and Clinic/ Study Centre
Comparable with the data, see the necessity that this also belongs to the processes of the biobank and handled separately from samples and data.
Ethical legal and social issues
General, overarching standards for biobanks
Patient information
From my point of view, a rough overview would be sufficient
Legal aspects</td></tr>
<tr><td colspan="2">If you have other topics that you consider important with regards to further training, you can list them here:</td></tr>
<tr><td>Technical personnel</td><td>Co-Workers of the technical personnel</td></tr>
<tr><td>Use of technical devices
Or annual safety instructions
Specialist knowledge about ischemic times, e.g. information about biological backgrounds etc. Medical information on tissue origin (anatomy etc.)
Improvement DNA/RNA storage
English
Research results on sample storage, who is allowed to inform patient, IC?</td><td>Infection protection
Experience with common biobank software
LIMS/ BIMS
Documentation of biospecimens! In general the knowledge why data should be stored structured (for computer scientists: atomic/in normal form) in a database and not in free text is missing.
Project management, time management</td></tr>
</table>
